# Genes Influence Young Children’s Human Figure Drawings and Their Association With Intelligence a Decade Later

**DOI:** 10.1177/0956797614540686

**Published:** 2014-10

**Authors:** Rosalind Arden, Maciej Trzaskowski, Victoria Garfield, Robert Plomin

**Affiliations:** 1 MRC Social, Genetic and Developmental Psychiatry Centre, Institute of Psychiatry, King’s College London; 2 Department of Epidemiology and Public Health, University College London

**Keywords:** cognitive ability, cognition(s), creativity

## Abstract

Drawing is ancient; it is the only childhood cognitive behavior for which there is any direct evidence from the Upper Paleolithic. Do genes influence individual differences in this species-typical behavior, and is drawing related to intelligence (*g*) in modern children? We report on the first genetically informative study of children’s figure drawing. In a study of 7,752 pairs of twins, we found that genetic differences exert a greater influence on children’s figure drawing at age 4 than do between-family environmental differences. Figure drawing was as heritable as *g* at age 4 (heritability of .29 for both). Drawing scores at age 4 correlated significantly with *g* at age 4 (*r* = .33, *p* < .001, *n* = 14,050) and with *g* at age 14 (*r* = .20, *p* < .001, *n* = 4,622). The genetic correlation between drawing at age 4 and *g* at age 14 was .52, 95% confidence interval = [.31, .75]. Individual differences in this widespread behavior have an important genetic component and a significant genetic link with *g*.

In 1926, a young woman struggled to come up with a reliable way to measure the intelligence of young children. Florence Goodenough (1886–1959) conceived the idea of asking the children to draw a human figure. Her ability test was remarkable: It took 10 min or fewer to administer; it used cheap, familiar, and widely available materials; children enjoyed the task; and the test could be scored easily and reliably (Brill, 1935; Goodenough, 1926; Oakland & Dowling, 1983). Crucially, it worked: Performance on the Goodenough Draw-a-Man Test correlated moderately with scores on time-consuming comprehensive IQ tests, and the test was both reliable and valid (Abell, Wood, & Liebman, 2001; Naglieri & Maxwell, 1981). Goodenough’s genius was to take a common childhood product and see its potential as an indicator of cognitive ability (Abell et al., 2001; Chambers, 1983; Chappell & Steitz, 1993; Jones & Rich, 1957; Krohn & Traxler, 1979; Plubrukarn & Theeramanoparp, 2003). The test was validated in several populations and used widely until its popularity declined in the 1970s, perhaps because it was considered by some researchers to be one of several projective techniques, including the Rorschach Test, that were not empirically well supported (Lilienfeld, Wood, & Garb, 2000) for screening psychopathology (Chapman & Chapman, 1967), which was not its original purpose. The test has not previously been analyzed in genetically informative samples, so the etiology of individual differences in children’s figure drawing is unsettled.

Behavioral genetic designs, such as the twin design comparing resemblance between identical (monozygotic, or MZ) twins and fraternal (dizygotic, or DZ) twins, are especially interesting to apply to differences in children’s drawings of human figures because such drawings seem so likely to be sensitive to family background, such as parental guidance and encouragement. It also seems intuitive that any relationship between early figure drawing and later intelligence would be caused by familial influences held in common between the two traits. It seems that children with ready access to pencils, paper, picture books, and so on would have better drawing skills than children brought up without those advantages. These credible scenarios can be tested empirically with a twin study (Plomin, DeFries, Knopik, & Neiderhiser, 2013).

Another reason to examine drawing is that it is ancient and widespread; cave decorations have been dated to 40,000 years ago (Pike et al., 2012; Valladas et al., 1992). Humans’ adult ancestors sculpted clay models of the human figure and then fired the figurines in ash pits 14,000 years before making “useful” artifacts such as pots. Evidence that figurative drawing and sculpture were valued comes from the number of hours spent in creating them, and from the places within cave sites where the objects were stored (Cook, 2013, p. 35). Behavior rarely fossilizes, yet it has been preserved, marvelously, in fresh and beautiful drawings (and sculptures) in places like the caves of Chauvet and Lascaux (Bataille, 1955; Chalmin et al., 2004; Chauvet, Brunel, & Hillaire, 1995, p. 114), and no doubt many as-yet-undiscovered sites, given the paucity of the record (Cook, 2013).

Here we report our findings from the first genetically informative study of individual differences in children’s figure drawings and their relation to intelligence measured a decade later. We aimed to discover (a) the extent to which (if any) genes influence individual differences in children’s drawings of human figures, (b) the extent to which the accuracy of such drawings is predictive of later intelligence, and (c) the extent to which genes that contribute to drawing at age 4 also contribute to intelligence up to a decade later.

## Method

### Sample

Our sample comprised twins from the Twins Early Development Study (TEDS; Haworth, Davis, & Plomin, 2013). The Office for National Statistics, on behalf of the study, contacted all families with live twin births in England and Wales from 1994 through 1996. Although there has been some attrition, comparisons with the general population show that families in TEDS remain closely representative of the British population in socioeconomic distribution, ethnicity, and parental occupation (Haworth et al., 2013). Their average intelligence resembles that of the general population; their range of intelligence extends to 3.5 standard deviations away from the mean in both directions. Our sample varied in size for different ages and trait measurements because not every TEDS family participates in every wave of data collection. The total sample used for our full-information maximum-likelihood analyses consisted of 7,752 twin pairs; of these, 7,437 pairs contributed data on drawing at age 4, 7,231 pairs contributed data on *g* at age 4, and 2,348 pairs contributed data on *g* at age 14. Sample sizes by age, trait, and zygosity are given in full in Table S3 in the Supplemental Material available online. Zygosity was assigned following a parent-administered questionnaire with 95% accuracy compared with DNA markers (Price et al., 2000); uncertain cases were resolved with DNA markers. We included same-sex and opposite-sex twins in our analyses after testing for sex-specific effects (see Analyses).

### Measures

#### Drawing

When the twins were 4 years old, their parents received a questionnaire that included the Draw-a-Child test (McCarthy, 1972). The drawing test was administered separately to each twin by a parent. Each child drew on the same size and type of paper (a booklet was provided by the study). The standardized instructions to the parent were as follows:

If your child is a girl, say: “Draw me a picture of a girl. Do the best that you can. Make sure that you draw all of her.” If your child is a boy, say: “Draw me a picture of a boy. Do the best that you can. Make sure that you draw all of him.” If your child hesitates, encourage him/her, saying things like “You draw it all on your own, and I’ll watch you. Draw the picture any way you like, just do the best picture you can.” Do not say which parts of the body to draw. It is very important that you do not mention any of the body parts that your child could include in the picture. If your child stops before the picture seems to be finished, say “Is s/he finished? Is that all of him/her?” When your child has finished the picture be sure to have a look at it, and admire it!

The scoring for the Draw-a-Child test is straightforward and objective. A drawing receives 1 point for the presence and correct quantity of each of the following bodily features: head, eyes, nose, mouth, ears, hair, body, arms, legs, hands, and feet. For example, a frontal drawing of a figure showing two legs, rather than four or none (4-year-old children rarely draw in profile; Cox, 1993, p. 58), would score 1 point for legs. Any clothing indicated on the drawing scores 1 point. Thus, scores range from 0 to 12. This scoring system ignores features such as overall size, charm, proportion, expressed emotion, whether circles are closed or open, and other characteristics of children’s drawings. Published interrater reliability for this test is .93 (Naglieri & Maxwell, 1981). The internal consistency of the test (Cronbach’s α), calculated empirically from our sample, was .79. Published test-retest (1-month) stability coefficients for this test, combined with another test of perceptual performance, were .78 in 3-year-olds and .84 in 5-year-olds (McCarthy, 1972, p. 34).

#### Intelligence

At ages 4 and 14, the children were administered verbal and nonverbal tests of cognitive ability (Oliver & Plomin, 2007; Spinath, Ronald, Harlaar, Price, & Plomin, 2003). At age 4, these tests comprised a sentence-construction test derived from the MacArthur Communicative Development Inventory adapted for the United Kingdom (Fenson, Pethick, & Cox, 1994) and nonverbal tests: an odd-one-out test, a design drawing test, a puzzle test, and 12 items testing conceptual knowledge (taken from the hour-long Parent Report of Children’s Abilities; Price, Eley, Dale, Stevenson, & Saudino, 2000). At age 14, the children were administered (over the Web) 30 items from Raven’s Standard Progressive Matrices (Raven, Court, & Raven, 1996) and a 27-item vocabulary test from the Wechsler Intelligence Scale for Children (Kaplan, Fein, Kramer, Delis, & Morris, 1999).

At each age, we used a unit-weighted composite of the verbal and nonverbal test scores (after scores were residualized against age and sex and then standardized) as an index of intelligence. For analysis, all verbal and nonverbal scores were transformed using rank-based quantile normalization (Lehmann, 1975), as in other reports on this sample (Hanscombe et al., 2012).

### Analyses

We calculated descriptive statistics to examine the distribution of our key measures and conducted analyses of variance (ANOVAs) to probe the effects of sex and zygosity on the means and standard deviations of those measures. We calculated phenotypic correlations within a twin model using OpenMx (Boker et al., 2011) to capitalize on the method of full-information maximum likelihood.

Univariate analyses were conducted using OpenMx. An introduction to the principles, methods, and assumptions of biometric models is given in Medland and Hatemi (2009). The univariate *ACE* model depends on known differential genetic relatedness within a sample: DZ twins share half their segregating genes, whereas MZ twins share them all. This difference, together with variance and covariance of the observations, is used to estimate the relative influence of additive genetic effects (*A*), shared (or common) environmental differences (*C*), and nonshared (or individual-specific) nongenetic differences (*E*). The *ACE* model assumes equality of means and variances across groups; we tested this assumption (results not reported) by examining a model in which the means and variances across twin groups^1^ and zygosity groups were constrained to be equal. If this model fit the data significantly worse than an unconstrained model, that would be evidence against the assumptions of this model for these data. It is usual to compare the fits of the alternative model (*ACE*) and the baseline (saturated) model. In addition, we tested for qualitative and quantitative sex differences using a sex-limitation model. We report the heritability estimates (with confidence intervals, or CIs) of drawing scores, *g* at age 4, and *g* at age 14.

In trivariate analyses, we used a Cholesky decomposition to estimate the genetic and environmental contributions to the covariance between *g* at age 4, drawing at age 4, and *g* at age 14. These analyses decompose the covariance among traits to estimate the contributions from genes and the shared environment. Measurement error and unique factors that may influence traits do not usually contribute to covariance because errors and stochastic events tend to be uncorrelated. All these analyses were conducted in OpenMx. The covariance of drawing at age 4 and *g* at age 14 was our focal interest, but because we expected that *g* at age 4 would be phenotypically correlated with drawing at age 4, we included *g* at age 4 in the model.

## Results

**Fig. 1.**
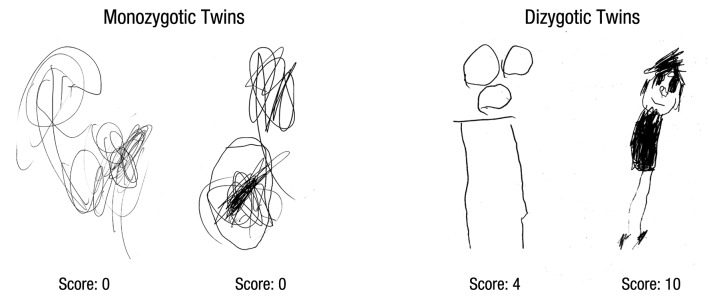
Sample drawings of one pair of monozygotic twins (left) and one pair of dizygotic twins (right), with the scores the drawings received.

Figure 1 shows the drawings of one pair of MZ twins and one pair of DZ twins. These sample drawings are illustrative of our quantitative findings described in this section.

### Descriptive statistics

Raw figure-drawing scores (*N* = 7,752 pairs of twins) ranged from 0 to 12 (*M* = 6.78, *SD* = 2.77). Measures of *g* were standardized from the whole sample at each age; among the children included in our analyses, means at age 4 and 14 were approximately 0, and standard deviations were approximately 1. Untransformed figure-drawing scores and *g* were both distributed normally. ANOVAs (see Table S1 in the Supplemental Material) showed significant effects of both sex and zygosity on the key variables (as expected in this very large sample), but the *R*^2^ values (range = .006–.07) showed that the interactive and the main effects of sex and zygosity were fairly small. We observed a slight difference between MZ and DZ twins in the variance of our cognitive-ability measures, which we attribute to our large sample size. (See Tables S1, S4, and S5 and Figs. S1 and S2 in the Supplemental Material for all means and variances, descriptive statistics by sex and zygosity, and histograms showing the distribution of raw drawing scores and age- and sex-corrected drawing and *g* scores.)

### Phenotypic correlations between drawing and intelligence

Table 1 shows that scores on the figure-drawing test at age 4 were phenotypically correlated with *g* measured at the same age, and also later at age 14. Surprisingly, the drawing scores correlated almost as much with *g* at age 14 as did *g* at age 4, which was measured from a larger test battery.

**Table 1.** Zero-Order Phenotypic Correlations Among the Key Measures (*N* = 7,752 pairs)

Variable	Drawing at 4	*g* at age 4
*g* at age 4	.33 [.32, .35]	
*g* at age 14	.20 [.17, .22]	.24 [.21, .27]

Note: The correlations and 95% confidence intervals, in square brackets, are maximum-likelihood estimates. All three correlations are significant, *p* < .001 (two-tailed).

### Intraclass correlations (ICCs)

We calculated ICCs to estimate whether genetic influence was exerted on the measured traits (see Table S2 in the Supplemental Material). If the ICCs suggested genetic influence, we would then fit the raw data to models to obtain more refined estimates of etiological influences. In our sample, the drawing and *g* scores of MZ twins were more similar to each other than were the scores of DZ twins. ICCs for the drawing scores were .55, 95% CI = [.53, .58], for MZ twins (*n* = 5,048) and .39, 95% CI = [.37, .42], for DZ twins (*n* = 9,826). The ICCs for *g* at age 4 were .88, 95% CI = [.87, .88], for MZ twins (*n* = 4,901) and .72, 95% CI = [.71, .73], for DZ twins (*n* = 9,560). ICCs for *g* at age 14 were .58, 95% CI = [.55, .60], for MZ twins (*n* = 1,812) and .35, 95% CI = [.33, .37], for DZ twins (*n* = 2,883). The ICCs suggested that genes exert an influence on both drawing and *g*.

### Univariate model fitting

We conducted univariate model fitting on *g* and drawing scores at age 4 and *g* at age 14. Additive genetics accounted for .29 of the variance in children’s drawing scores (95% CI = [.22, .35]), the shared environment accounted for .23 of the variance (95% CI = [.18, .29]), and unique nongenetic factors accounted for .51 of the variance (95% CI = [.48, .54]). The estimates of heritability for all three measured traits are shown in Table 2. Figure drawing at age 4 was as heritable as *g* at age 4 (.29, 95% CIs = [.22, .36] and [.27, .32], respectively). As has been reported previously, the heritability of *g* increased between ages 4 and 14. We found no sex differences in our sex-limitation models, so we combined data from boys and girls in our trivariate model.

**Table 2.** Heritability of the Key Measures (Estimated From Univariate Models)

Measure	Heritability
Drawing at age 4 (*n* = 14,874)	.29 [.22, .35]
*g* at age 4 (*n* = 14,461)	.29 [.27, .32]
*g* at age 14 (*n* = 4,695)	.50 [.38, .61]

Note: The values in square brackets are 95% confidence intervals.

### Trivariate model fitting

**Fig. 2.**
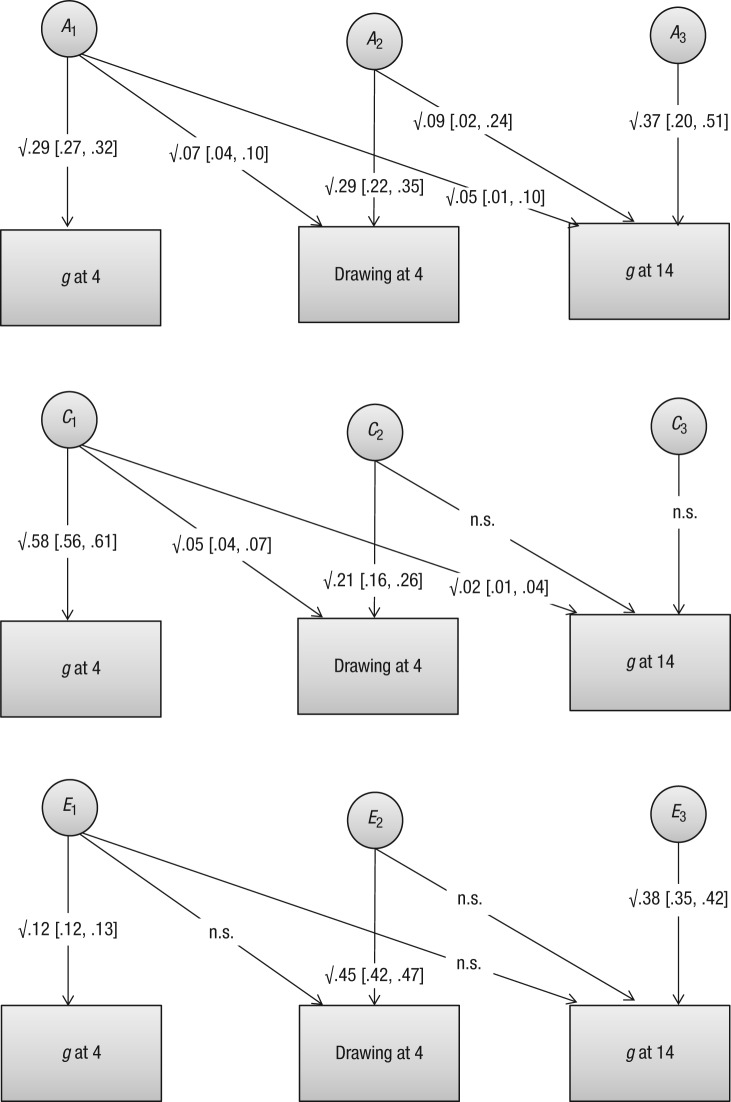
Trivariate Cholesky decomposition showing estimates (with 95% confidence intervals in brackets) of squared standardized path coefficients (proportion of variance accounted for) for additive-genetic (*A*), shared-environment (*C*), and stochastic or person-specific-environment (*E*) effects. The subscripts 1, 2, and 3 refer to *g* at age 4, drawing at age 4, and *g* at age 14, respectively.

Figure 2 shows point estimates of the proportion of variance explained, along with their 95% CIs. These coefficients are shown alongside arrows pointing from the variance components (*A, C*, and *E*) to the traits. The total genetic and nongenetic effects for each trait can be estimated by summing the contributions of the three components. Additive genetics (*A*) had the biggest systematic influence on differences in children’s drawing scores (.07 + .29 = .36). This estimate of heritability is slightly higher than that derived from the univariate analysis because genetic influence on drawing in the trivariate analysis included the genetic influence that drawing and *g* at age 4 held in common. The only systematic influence on the covariance between drawing at age 4 and *g* at age 14 was genetic, estimate = .09, 95% CI = [.02, .24]. The genetic correlation between figure drawing at age 4 and *g* at age 14 was .52, 95% CI = [.31, .75] (not shown in the figure). Figure 2 shows that although the shared environment (*C*) exerted a significant influence on drawing scores at age 4 (.05 + .21 = .26), its influence on *g* measured at age 14 dropped to zero.

We calculated various fit indices, including the Bayesian information criterion, which is less sensitive than other indices to large sample sizes. These statistics (see Table 3) showed a good fit for the trivariate model.

**Table 3.** Fit Statistics Comparing the Saturated Full Model With the Trivariate Cholesky Model

Model	–2LL	*df*	AIC	BIC
Saturated	85,530.89	33,976	17,578.89	–218,748.18
Trivariate	85,604.07	34,009	17,586.07	–218,970.54

Note: A chi-square test indicated that the fit of the trivariate Cholesky model was significantly better than the fit of the saturated model, χ^2^ = 73.18, *p* = .0000711. LL = log likelihood; AIC = Akaike’s information criterion; BIC = Bayesian information criterion. Smaller (more negative) BIC values indicate better fit.

Biometric models make assumptions about the data. These include the assumptions that zygosity has been correctly assigned, that the measures are multivariate normal, and that means and variances are equal between groups. In this large sample, there were small mean and variance differences between twin and zygosity groups, so we conducted formal tests to check that the deviations did not violate the model. These tests showed that a single mean and variance for each trait could be used in the model. We found that 99% of the phenotypic correlation between drawing at age 4 and *g* at age 14 (*r* = .20) was mediated genetically.

## Discussion

We found that drawings done by MZ twins were significantly more similar than were drawings done by DZ twins. Finding that a behavior is heritable is no longer news; yet if the data had shown that any siblings’ drawing scores were alike, irrespective of zygosity, we would not have been surprised because it seems so plausible that young same-age siblings would emulate each other’s drawings or be guided by parents (irrespective of zygosity). For that reason, we were intrigued to find that scores for a single drawing were as heritable as was *g* estimated from several different indicators (verbal and nonverbal tests). The high interrater reliability of the drawing test suggests that rater unreliability is unlikely to be the source of the individual-specific environmental influence on drawing at age 4 (*E*). In this large sample, a single picture of a 4-year-old child, drawn in around 5 min, had a significant positive phenotypic association with *g* measured a decade later, and this correlation was as high as the correlation between *g* at age 4 and *g* at age 14. This phenotypic association was caused partly by a genetic correlation between drawing at age 4 and *g* at age 14.

Our data show that the capacity to realize on paper the salient features of a person, in a schema, is an intelligent behavior at age 4. Performance of this drawing task relies on various cognitive, motoric, perceptual, attentional, and motivational capacities. Our estimated positive phenotypic correlation between drawing and contemporaneous intelligence is consistent with estimates from 40 small studies in which the correlations (*r*s) ranged from .24 to .83 (Scott, 1981; see Willcock, Imuta, & Hayne, 2011). The correlation we observed is also consistent with a large phenotypic study of 7-year-olds that found, perhaps surprisingly, that figure-drawing scores correlated with arithmetic performance (*r* = .33, *n* = 14,522) to about the same extent as they correlated with pattern copying (*r* = .37, *n* = 14,545; Shepherd, 2012, p. 21).

We do not know whether those children who scored higher on the drawing task at age 4 will be more likely to develop a sustained interest in art. This study does not explain artistic talent; the scores only quantify accuracy of attributes, such as the number of limbs, in the drawings. But our results do show that whatever conflicting theories adults have about the value of verisimilitude in early figure drawing, children who express it to a greater extent are somewhat brighter than those who do not.

This study had great statistical power, but any sample has some restrictions. For example, people at the extreme ends of various distributions (including social and economic distributions) are underrepresented in almost all studies. Also, our analyses were subject to the usual assumptions of the twin method, which have been explored elsewhere (see Plomin et al., 2013).

There is some evidence in the archaeological record that figurative art is more recent than geometric patterning (Pike et al., 2012). If this is correct, then figurative art may track, to some extent, increasing cognitive ability in the human species. Drawing is an ancient human capacity; 32,000 years before the children in our study sat down to draw, unknown people made surviving drawings of great skill and beauty. These images (see Bradshaw Foundation, 2011, for photographs) are among the oldest examples of a human behavior that continues in the same form today. This long history endows the drawing test with ecological validity and relevance to an extent that is unusual in psychometrics.

Drawing marks called finger flutings, made by dragging fingers across wet clay or on soft cave walls, are the oldest known direct evidence of children’s behavior, aside from footprints. Archaeologists have dated these marks to the Upper Paleolithic and ascribed them to young children on the basis of detailed measurements of the groove widths (Sharpe & Van Gelder, 2006). The longevity of children’s drawing behavior indicates that drawing is a natural part of the human species-typical repertoire. Given that drawing enhances the fine-motor skills that children use in writing (Saida & Miyashita, 1979), it may have contributed to the development of pictograms, and eventually writing.

The finding that greater accuracy in children’s figure drawing is associated genetically with higher *g* a decade later is thought provoking and demonstrates that the study of art and the study of science have much to offer each other. Evolutionary selection on drawing ability may have been an important precursor to writing, which transformed humans’ capacity to store information externally, and promoted the capacity to build a civilization.

## Notes

1. In each twin pair, one twin was assigned to be Twin 1 and the other to be Twin 2. We refer to Twins 1 and Twins 2 as twin groups.
